# Public Reactions to the New York State Policy on Flavored Electronic Cigarettes on Twitter: Observational Study

**DOI:** 10.2196/25216

**Published:** 2022-02-03

**Authors:** Li Sun, Xinyi Lu, Zidian Xie, Dongmei Li

**Affiliations:** 1 Goergen Institute for Data Science University of Rochester Rochester, NY United States; 2 Department of Clinical & Translational Research University of Rochester Medical Center Rochester, NY United States

**Keywords:** New York State policy, flavored e-cigarettes, Twitter, social media, vaping, e-cigarette

## Abstract

**Background:**

Flavored electronic cigarettes (e-cigarettes) have become popular in recent years, especially among youth and young adults. To address the epidemic of e-cigarettes, New York State approved a ban on sales of most flavored vaping products other than tobacco and menthol flavors on September 17, 2019.

**Objective:**

This study aims to examine the attitude of Twitter users to the policy on flavored e-cigarettes in New York State and the impact of this policy on public perceptions of e-cigarettes. This study also compares the attitudes and topics between New York Twitter users and Twitter users from other states who were not directly affected by this policy.

**Methods:**

Tweets related to e-cigarettes and the New York State policy on flavored e-cigarettes were collected using the Twitter streaming application programming interface from June 2019 to December 2019. Tweets from New York State and those from other states that did not have a flavored e-cigarette policy were extracted. Sentiment analysis was applied to analyze the proportion of negative and positive tweets about e-cigarettes or the flavor policy. Topic modeling was applied to e-cigarette–related data sets and New York flavor policy–related data sets to identify the most frequent topics before and after the announcement of the New York State policy.

**Results:**

We found that the average number of tweets related to e-cigarettes and the New York State policy on flavored e-cigarettes increased in both New York State and other states after the flavor policy announcement. Sentiment analysis revealed that after the announcement of the New York State flavor policy, in both New York State and other states, the proportion of negative tweets on e-cigarettes increased from 34.07% (4531/13,299) to 44.58% (18,451/41,390) and from 32.48% (14,320/44,090) to 44.40% (64,262/144,734), respectively, while positive tweets decreased significantly from 39.03% (5191/13,299) to 32.86% (13,601/41,390) and from 42.78% (18,863/44,090) to 33.93% (49,105/144,734), respectively. The majority of tweets related to the New York State flavor policy were negative both before and after the announcement of this policy in both New York (87/98, 89% and 3810/4565, 83.46%, respectively) and other states (200/255, 78.4% and 12,695/15,569, 81.54%, respectively), while New York State had a higher proportion of negative tweets than other states. Topic modeling results demonstrated that teenage vaping and health problems were the most discussed topics associated with e-cigarettes.

**Conclusions:**

Public attitudes toward e-cigarettes became more negative on Twitter after New York State announced the policy on flavored e-cigarettes. Twitter users in other states that did not have such a policy on flavored e-cigarettes paid close attention to the New York State flavor policy. This study provides some valuable information about the potential impact of the flavored e-cigarettes policy in New York State on public attitudes toward flavored e-cigarettes.

## Introduction

Tobacco smoking is a well-known risk factor for many diseases such as heart disease, cancer, and pulmonary disease [1]. Since flavored tobacco products attract people by hiding the natural harshness and taste of tobacco, the US Food and Drug Administration banned the sale of candy- and fruit-flavored cigarettes in 2009 [1,2]. Cigarette smoking among youth has declined in recent years, but the usage of electronic cigarettes (e-cigarettes) especially among youth has increased dramatically in recent several years [3]. Since 2014, e-cigarettes have become the most commonly used tobacco product among the youth [4]. The Centers for Disease Control and Prevention has reported that between 2011 and 2015, the usage of e-cigarettes has increased by more than 800% among middle school and high school students [5]. The 2019 National Youth Tobacco Survey data has shown that 27.5% of high school students and 10.5% of middle school students are currently using e-cigarettes [4].

Similar to flavored cigarette, flavored e-cigarette attracts people by its affordability, accessibility, convenience, and more importantly, a variety of flavors [6]. A study based on Population Assessment of Tobacco and Health Study (PATH) Wave 3 data showed that the most popular e-cigarette flavors are fruit and candy [7]. However, recent studies showed that flavored e-cigarettes could be harmful to lung tissues by imposing oxidative stress and inflammatory responses [8]. It is well known that e-cigarettes release volatile carbonyls, furans, nickels, lead, and chromium, which may be poisonous to the lungs [9]. In addition, e-cigarettes could harm endothelial cells that line the interior of human blood vessels and may increase the risk of heart disease [10]. The number of reported cases of e-cigarette or vaping use–associated lung injury (EVALI) increased rapidly in the United States in 2019 [11]. As of October 8, 2019, there were 1299 cases reported to the Centers for Disease Control and Prevention [12], and as of January 14, 2020, the number increased to 2668 [11]. Among patients with EVALI, 76% are younger than 35 years [11].

Owing to the potential negative health effects of flavored e-cigarettes, starting from June 2019, many states and cities in the United States have announced the ban on flavored e-cigarettes. On June 25, 2019, San Francisco became the first US city to ban the sale and distribution of e-cigarettes in the city [13]. Michigan (starting from September 4, 2019) and New York (starting from September 17, 2019) announced the policy regulating the sales of most flavored vaping products [14,15]. Following New York State, Rhode Island, Los Angeles County, Oregon State, Montana State, Washington State, New Jersey, and Massachusetts passed the ban on the sale of flavored vaping products [15-22].

Many recent e-cigarettes studies have utilized social media data to identify topics related to e-cigarettes. For example, Kavuluru and Sabbir [23] developed a supervised predictive model to identify e-cigarette proponents on Twitter. Zhou et al [24] investigated the influence of flavors on the propagation of e-cigarette–related information on Facebook. As one of the most popular social media platforms in the United States, Twitter contains many e-cigarette–related posts (tweets), which provides us an ideal avenue to investigate the public opinion on the policies regulating flavored e-cigarettes. In addition, messages from social media can influence people’s attitudes and behaviors [25]. Pew Research Center found that approximately 20% of social media users might change their opinions after they view related messages on social media [25]. According to Pew Research Center data in 2019, compared to the whole population, Twitter users are younger, which corresponds to the potential users of e-cigarettes [26]. Compared to national surveys such as the PATH studies that have been used to study public opinions on tobacco products, social media studies could provide immediate reactions to policy shifts, larger sample size, much less data collection cost, and less biased responses [27].

Previous studies have investigated public attitudes toward e-cigarette regulation and policy by using sentiment analysis. The results showed that regulation was considered as a fundamental requirement for public health protection [28]. Instead of supporting a blanket ban on public vaping due to the perceptions of insufficient evidence on the harm of vaping, the study participants supported the right for individuals and organizations to restrict vaping [28]. The public attitudes toward the health policy and especially e-cigarettes will potentially affect user behavior, which is the primary goal of these health policies. Since flavored e-cigarettes policy was announced in the United States, it was important to understand the public attitudes toward the policy and how the public might react to the policy.

In this study, we aimed to investigate public responses toward the New York State flavor policy on Twitter by applying sentiment analysis and topic modeling to related tweets before and after the announcement of the policy. Furthermore, we compared the sentiments and topics from New York State and other states that did not have a flavored e-cigarette policy to examine the potential impact of the flavor policy in New York State on public attitudes toward e-cigarettes. We hypothesized that the public attitude toward flavored e-cigarettes would become more negative after the announcement of the flavor policy in both New York State and other states.

## Methods

### Data Collection and Preprocessing

e-Cigarette–related Twitter posts between June 2019 and December 2019 were downloaded from the Twitter streaming application programming interface by using e-cigarette–related keywords. The e-cigarette–related keywords include e-cig, e-cigs, ecig, ecigs, electroniccigarette, ecigarette, ecigarettes, vape, vapers, vaping, vapes, e-liquid, ejuice, eliquid, e-juice, vapercon, vapeon, vapefam, vapenation, and juul [29-31]. To avoid the potential impact of related flavor ban information posted on Twitter right before the announcement of the New York State flavor policy on September 17, 2019, we excluded the tweets posted from September 1 to September 16, 2019. Meanwhile, to avoid the potential overreaction of e-cigarette users to the New York State flavor policy immediately after its announcement and to examine more logical responses to the New York State flavor policy, we excluded tweets posted from September 17 to September 30, 2019 in our study.

To remove the promotion tweets, we filtered out Twitter IDs that contained promotion-related keywords (such as dealer, deal, store, supply, e-cig, store, promo, and promotion) [31]. In addition, we filtered out Twitter posts that contained promotion-related keywords (dealer, deal, customer, promotion, promo, promos, discount, sale, free shipping, sell, $, %, dollar, offer, percent off, store, save, price, wholesale) [31]. After these 2 filtering steps, 2 data sets with e-cigarette–related tweets were created based on the posted date. One data set included e-cigarette tweets between June 13, 2019 and August 22, 2019, which is considered as before New York State announced the policy on flavored vaping products. The other data set was between October 1, 2019 and December 31, 2019, which is regarded as after New York State announced the policy. The first data set contained 724,345 e-cigarette tweets, and the second one contained 2,130,748 e-cigarette tweets. The number of unique users was identified based on the Twitter user ID. The first data set contained 599,146 unique users, and the second one contained 680,967 unique users.

We further extracted tweets related to the New York State policy on flavored e-cigarettes by filtering with keywords “ban” and “bans.” To ensure that these tweets were about the policy in New York State, we eliminated the tweets that mentioned other states with a ban on flavored e-cigarettes but not mentioned New York State. In total, we collected 68,318 New York State flavor policy–related tweets from June 2019 to December 2019, which included 353 before the policy and 20,134 tweets after the announcement of the New York flavor policy.

For both e-cigarette–related and New York flavor policy–related data sets, we applied 2 state-filtering processes on the geotagged tweets or the users who included the location information in the profile metadata to derive a New York State subset and other states subset (without a ban on flavored e-cigarettes) as the control group. First, we filtered the data sets by keywords “ny” and “new york” on the location of the user and the place of the tweets, which is the New York State subset. Second, we used the same procedure to filter the data sets with keywords “usa,” “united states,” and “us” to extract US tweets, and then, we eliminated the tweets from San Francisco, Michigan, New York State, Rhode Island, Los Angeles County, Oregon State, Montana State, Washington State, New Jersey, and Massachusetts that have policies on flavored e-cigarettes, which is the data set for other states.

### Sentiment Analysis

The Valence Aware Dictionary and sEntiment Reasoner was used as the sentiment analyzer to extract Twitter users’ opinions on e-cigarettes and New York State flavor policy [32]. For both e-cigarette–related data sets and New York State flavor policy data set, we calculated the sentiment scores for each tweet. Tweets with sentiment scores between –1.00 and –0.05 were classified as negative tweets, tweets with scores between –0.05 and +0.05 (not including –0.05 and 0.05) were classified as neutral tweets, and tweets with scores between +0.05 and +1.00 were classified as positive tweets. To better compare the differences between different states and periods, we normalized the number of negative, neutral, and positive tweets by the total number of tweets in different states in each period. Finally, we conducted 2-sided 2-proportion z-tests to test whether the proportions of negative and positive tweets between New York State and other states were significantly different.

### Topic Modeling

The Latent Dirichlet Allocation model was conducted on e-cigarette–related data sets to extract the most frequently discussed topics. Latent Dirichlet Allocation, a type of topic modeling algorithm, is an unsupervised learning model that gives the number of topics, assigns each word in the document to a specific topic, and calculates a weight for each word in every topic [33]. First, after removing all punctuations and converting all texts to lowercase, we tokenized every sentence. Second, we applied the Natural Language ToolKit package to remove stop words (eg, the, a, in). Third, we used the Genism package to convert some frequent bigrams and trigrams to a single term. At last, we lemmatized all texts by implementing spaCy by changing all tenses to present tense and keeping only nouns, adjectives, verbs, and adverbs. To identify the optimal number of topics, we calculated the coherence scores that measure the relative distance between words within a topic. The number of topics was chosen based on the coherence scores. We selected the number of topics based on the maximum coherence score.

## Results

### Tweets Related to e-Cigarettes and the New York Flavor Policy on e-Cigarettes

We observed that in New York State or other states without any flavor policy, there was a significant increase in the percentage of daily tweets related to the flavor policy after the announcement of the New York flavor policy (Table 1). For the tweets related to the flavor policy, 0.74% (98/13,299) of all e-cigarette tweets were from New York State and 0.58% (256/44,090) of all e-cigarette tweets were from other states before the New York State flavor policy was announced. After the New York State flavor policy was announced, 10.93% (4565/41,764) of all e-cigarette tweets were related to the flavor policy from New York State and 11.11% (16,083/144,734) of all e-cigarette tweets were related to the flavor policy from other states.

**Table 1:** Proportion of tweets related to the New York flavor policy before and after its announcement in New York State and other states.

Time, state		Tweets, n (%)	
**Before the New York flavor policy**			
	In New York State (n=13,299)		98 (0.74)
	In other states (n=44,090)		256 (0.58)
**After the New York flavor policy**			
	In New York State (n=41,764)		4565 (10.93)
	In other states (n=144,734)		16,083 (11.11)

### Public Attitudes Toward e-Cigarettes on Twitter

To examine whether there was any change in the public opinions toward e-cigarettes with the announcement of the New York flavor policy on e-cigarettes, we compared the proportions of negative, positive, and neutral tweets between before and after the announcement of the New York flavor policy in New York State and other states (Table 2 and Table 3, respectively). We observed that in both New York State and other states, compared to the period before the announcement of the New York flavor policy, the proportion of positive tweets on e-cigarettes significantly decreased (*P*<.001) after the New York flavor policy from 39.03% (5191/13,299) to 32.86% (13,601/41,390) and from 42.78% (18,863/44,090) to 33.93% (49,105/144,734) respectively. In contrast, the proportion of negative tweets related to e-cigarettes significantly increased (*P*<.001) after the New York State flavor policy announcement in New York State (from 4531/13,299, 34.07% to 18,451/41,390, 44.58%) and in other states (from 14,320/44,090, 32.48% to 64,262/144,734, 44.40%).

In both periods, the proportion of positive tweets in other states was significantly higher than that in New York State (18,863/44,090, 42.78% vs 5191/13,299, 39.03% before the policy; 49,105/144,734, 33.93% vs 13,601/41,390, 32.86% after the policy). Comparing the proportion of negative tweets in other states, before the New York State flavor policy was announced, New York State had a significantly higher (*P*<.001) proportion of negative posts (4531/13,299, 34.07% vs 14,320/44,090, 32.48%). However, after the announcement of the New York State flavor policy, there was no significant difference (18,451/41,390, 44.58% vs 64,262/144,734, 44.40%).

**Table 2:** Proportion of electronic cigarette–related tweets with different sentiments before and after the New York flavor policy announcement in New York State.

Time, sentiments			Tweets, n (%)
**Before the New York flavor policy (n=13,299)**			
	Negative	4531 (34.07)	
	Positive	5191 (39.03)	
	Neutral	3577 (26.90)	
**After the New York flavor policy (n=41,390)**			
	Negative	18,451 (44.58)	
	Positive	13,601 (32.86)	
	Neutral	9338 (22.56)	

**Table 3:** Proportion of electronic cigarette–related tweets with different sentiments before and after the New York flavor policy announcement in other states.

Time, sentiments			Tweets, n (%)
**Before the New York flavor policy (n=44,090)**			
	Negative	14,320 (32.48)	
	Positive	18,863 (42.78)	
	Neutral	10,907 (24.74)	
**After the New York flavor policy (n=144,734)**			
	Negative	64,262 (44.40)	
	Positive	49,105 (33.93)	
	Neutral	31,367 (21.67)	

### Public Attitudes Toward the New York State Flavor Policy on Twitter

To examine public attitudes toward the flavor policy on e-cigarettes, we conducted a sentiment analysis on the tweets related to the flavored e-cigarettes policy. As shown in Table 4 and Table 5, we observed that the majority of tweets related to the New York flavor policy were negative in both New York State (from 87/98, 89% to 3810/4565, 83.46%) and other states (200/255, 78.4% to 12,695/15,569, 81.54%). There was no significant change in the proportion of either positive or negative tweets between before and after the New York flavor policy in either New York State or other states.

We conducted 2 proportion z-tests to compare the proportion of tweets with different sentiments toward the flavor policy between the New York State and other states. In both time periods, New York State had a significantly higher proportion of negative tweets than other states (*P*=.03 before the policy, *P*=.003 after the policy). There was no significant difference in the proportion of positive posts between the New York State and other states before the announcement of the New York State flavor policy (*P*=.21). However, after the announcement of the policy, the proportion of positive tweets in other states was significantly higher than that in New York State (*P*<.001).

**Table 4:** Proportion of tweets with different sentiments toward the flavor policy before and after the New York flavor policy announcement in New York State.

Time, sentiments			Tweets, n (%)
**Before the New York flavor policy (n=98)**			
	Negative	87 (88.78)	
	Positive	10 (10.20)	
	Neutral	1 (1.02)	
**After the New York flavor policy (n=4565)**			
	Negative	3810 (83.46)	
	Positive	650 (14.24)	
	Neutral	105 (2.30)	

**Table 5:** Proportion of tweets with different sentiments toward the flavor policy before and after the New York flavor policy announcement in other states.

Time, sentiments			Tweets, n (%)
**Before the New York flavor policy (n=255)**			
	Negative	200 (78.43)	
	Positive	39 (15.29)	
	Neutral	16 (6.27)	
**After the New York flavor policy (n=15,569)**			
	Negative	12,695 (81.54)	
	Positive	2551 (16.39)	
	Neutral	323 (2.07)	

### Top Topics Discussed on e-Cigarettes

To further understand how the New York State flavor policy affected the public attitudes toward e-cigarettes, top topics related to e-cigarettes were generated before and after the announcement of the New York State flavor policy in New York (Table 6) and other states (Table 7). We observed that before the announcement of the New York State flavor policy, the topics about e-cigarettes between New York State and other states were similar. The majority of tweets focused on health or teenager vaping–related topics. For example, in both New York State and other states, a typical tweet is “Juul has created nicotine addiction in a whole generation of people who were statistically unlikely to start smoking cigarettes in the first place.”

After the announcement of the New York State flavor policy, we observed that while people kept discussing teenage vaping and smoking-related topics, the proportion of topics related to the policy increased, while the proportion of topics related to health decreased in both New York and other states. In addition, other states without a flavor policy had a higher proportion of topics related to the flavor policy compared to the New York State. The keyword “ban” appeared in the third topic in New York State while it showed up in the first and third topics in other states.

To examine whether there were some changes in the discussion about the New York flavor policy, top topics that related to New York flavor policy were generated before and after the announcement of the flavor policy in New York (Table 8) and other states (Table 9). Before the announcement of the New York flavor policy, the majority of the tweets focused on discussions about banning flavored e-cigarette products in both New York State and other states. However, after the New York flavor policy was announced, the topics were more diverse. Besides the discussions on banning flavored e-cigarettes, there were some discussions about teenager vaping and health problems, especially in New York State. Comparing to the topics from New York State after the announcement of the policy, the topics in other states tended to focus more on the policy of the flavored vaping products.

**Table 6:** Top topics related to electronic cigarettes discussed in New York State.

Topics		Token, n (%)	Keywords
**Before the New York flavor policy (n=13,299)**			
	Vaping leads to nicotine addiction in those who are unlikely to smoke	3524 (26.50)	cigarette, create, generation, addiction, smoking, first, start, whole, unlikely, statistically
	Lung diseases linked to vaping	2248 (16.90)	vape, stare, stupid_face, say, people, link, lung, case, teen, tell
	Quit smoking and vaping	2061 (15.50)	vape, go, make, think, smoking, quit, thing, vaping, year, smoker
	Juul gets good kids ill	2021 (15.20)	juul, hit, pod, look, get, good, kid, buy, illness, day
	Vaping leads to diseases and hospitalization	1942 (14.60)	use, friend, vaping, vape, beer, dear, disease, hospitalize, almost, call
	Ban cigarettes	1503 (11.30)	find, help, product, state, cigarette, would, report, add, ban, cig
**After the New York flavor policy (n=41,390)**			
	Teenager vaping	12,127 (29.30)	vape, vaping, say, go, want, kid, think, single, teen, see
	Smoking and vaping	11,920 (28.80)	vape, cigarette, vaping, get, smoking, people, consider, product, tobacco, age
	Ban flavored electronic cigarettes	7285 (17.60)	ban, flavor, let, vape, next, public, cig, pick, homeless, cup
	A joke about juul is cool like a refrigerator	5091 (12.30)	smoke, juul, year, thank, day, refrigerator, beaesg, mad, man, easy
	Discussion about flavored electronic cigarette policy	4925 (11.90)	juul, pod, government, impeach, formal_warn, flavor, look, hit, take, guy

**Table 7:** Top topics related to electronic cigarettes discussed in other states.

Topics			Token, n (%)		Keywords
**Before the New York flavor policy (n=44,090)**					
	Vaping leads to nicotine addiction in those who are unlikely to smoke	11,463 (26)		smoking, cigarette, generation, whole, addiction, start, first, unlikely, statistically, find	
	Teenage vaping juul	7628 (17.30)		juul, vape, hit, be, get, people, pod, kid, say, think	
	Lung diseases linked to vaping	7495 (17)		vape, go, stupid, face, lung, material, year, would, bad, cbd	
	Vaping among friends	7231 (16.40)		vape, new, use, friend, level, baby, stare, dear, stop, state	
	Health problems associated with teenager vaping	5820 (13.20)		create, vaping, smoke, link, cigarette, teen, damage, add, cig, health	
	Vaping is bad	4409 (10)		juul, case, meanwhile, chad, look, black, vaper, fuck, almost, rip	
**After the New York flavor policy (n=144,734)**					
	Discussions about the policy on banning flavored electronic cigarettes	54,999 (38)		vape, flavor, government, smoking, age, start, thank, product, warning, ban	
	Death associated with vaping	35,315 (24.40)		vape, vaping, beaesg, change, look, kill, fast, seem, teen, industry	
	Discussion on banning vaping	32,131 (22.20)		ban, next, single, consider, maybe, public, coffee, water, bottle, pick	
	Stop vaping juul	22,144 (15.30)		juul, smoke, pod, let, go, formal, fuck, ask, stop, bring	

**Table 8:** Top topics related to the New York flavor policy discussed in New York State.

Topic			Token, n (%)		Keywords
**Before the New York flavor policy (n=98)**					
	Ban flavored electronic cigarettes	52 (53)		ban, vaping, cigarette, vaping, ecig, product, smoking, smoker, would, mormon	
	Ban flavored electronic cigarettes, teenager vaping	46 (47)		ban, flavor, vape, kid, vaping, people, tobacco, cigarette, lead, young	
**After the New York flavor policy (n=4565)**					
	Ban flavored electronic cigarettes, teenager vaping	1278 (28)		ban, vape, vaping, flavor, product, cigarette, kid, nicotine, people, tobacco	
	Ban flavored electronic cigarettes, teenager vaping, health-related issue	1,173 (25.70)		ban, vape, flavor, teen, vaping, vaper, cig, health, people, tobacco	
	Discussion about the policy on banning flavored electronic cigarettes	1,137 (24.90)		ban, flavor, vape, trump, product, vaping, vote, say, go, back	
	Discussion about the policy on banning flavored electronic cigarettes	977 (21.40)		flavor, government, juul let, pod, impeach, formal, warning, information, false	

**Table 9:** Top topics related to the New York flavor policy discussed in other states.

Topics			Token, n (%)		Keywords
**Before the New York flavor policy (n=255)**					
	Ban flavored electronic cigarettes	147 (57.60)		ban, cigarette, smoker, hire, vape, smoke, quit, much, vaping, cig	
	Discussions about banning vaping	108 (42.40)		ban, vape, vaping, cig, juul, smoking, cigarette, smoke, mormon, public	
**After the New York flavor policy (n=15,569)**					
	Discussions about banning flavored vaping product	6071 (39)		ban, vape, product, flavor, vaping, trump, shop, industry, people, business	
	Discussions about banning flavored vaping product, death associated with vaping	4063 (26.10)		flavor, die, ban, vaping, vape, year, illegal, lead, hand, crisis	
	Discussions about the policy on banning flavored electronic cigarettes	3316 (21.30)		flavor, government, juul, let, pod, warning, impeach, formal, public, nicotine	
	Discussions about banning disguising vaping product	2119 (13.61)		vape, disguise, ban, see, maga_meh, away, show, school, listen, walk	

## Discussion

### Principal Findings

With the epidemic of e-cigarettes in the United States especially among youth and young adults, all tobacco regulatory policies aim to prevent the initiation of e-cigarettes use in youth. The New York State flavor policy was announced with the intention to protect youth, as flavors are one of the major reasons for the dramatic increase in youth vaping initiation and maintenance. Meanwhile, flavors are also key marketing strategies of vaping retailers and companies to attract youth to vape. Therefore, it is of utmost importance to evaluate the public perception of such a flavor policy, and more importantly, how the flavor policy affected the public perception of e-cigarettes, which might potentially affect user behavior to further protect public health, especially of the youth. We hypothesize that the New York State flavor policy will be supported by parents, health educators, and public health professionals and be opposed by current vapers or e-cigarette retailers or companies. To better test our hypothesis, we could distinguish individuals from organizations in future studies and examine the differences in sentiments and topics between individuals and organizations. This could help us explore and compare the attitudes of different groups of people.

In this study, we showed that after the announcement of the New York State flavor policy, the public attitudes to e-cigarettes became more negative in New York State and other states. In both New York State and other states, before the announcement of the New York State flavor policy, the greatest proportion of e-cigarette–related tweets was positive tweets, but after the policy was announced, the greatest proportion was negative tweets. One possible reason for more negative attitudes toward e-cigarettes could be the increased exposure of the public to the potential harm of vaping. Meanwhile, although the keyword “ban” was not be included in these tweets, it is possible that some tweets might be critical of the New York flavor policy, which could partially contribute to more negative attitude. Our results showed that the public attitudes toward the flavor policy on flavored e-cigarettes remained negative and did not change much between before and after the New York flavor policy in both New York State and other states. One possible explanation is that these Twitter users might be more likely to be e-cigarette users who want to continue vaping.

Although not statistically significant, we observed an increase in negative tweets related to the New York flavor policy in other states, which contrasted the decrease in negative tweets related to the New York flavor policy in New York after the New York flavor policy was announced. This might be because Twitter users in New York State might accept the policy after the New York flavor policy was announced while Twitter users in other states might worry that they would have a similar flavor policy in their states. As neither the changes in New York State nor other states were significant, the observed differences could also be due to random noise.

By applying topic modeling to examine the main topics related to e-cigarettes and the New York flavor policy after this policy announcement on flavored e-cigarettes, we showed that besides the discussion about the flavor ban, the main topics were teenage vaping and health-related, which might cause the increase in the proportion of negative tweets. In addition, these 2 topics were also mentioned frequently by Twitter users in New York State and other states before the New York State flavor policy was announced, which could due to the occurrence of EVALI in 2019. These topics showed public awareness of e-cigarettes’ harmfulness. In addition, we showed that other states had a higher proportion of tweets discussing the flavor ban after the policy in New York State was announced. These results suggest that Twitter users in the states that did not have a ban on flavored e-cigarettes had a significant concern about the potential regulatory policy on flavored e-cigarettes in their states.

### Comparison With Prior Work

Compared with that in a previous study analyzing e-cigarette tweets between October 2015 and February 2016 [34], the proportion of positive tweets toward e-cigarettes in our study decreased significantly. The percentage of positive tweets about e-cigarettes decreased from previously reported 66.4% (589/887) to 39.03% (5191/13,299) in New York State and 42.78% (18,863/44,090) in other states before the New York State policy on flavored e-cigarettes was announced, which might result from the epidemic of EVALI in 2019. After the New York State flavor policy was announced, the proportion of positive tweets on e-cigarettes was even lower. One previous study showed that although the prevalent topics were about the stigma and the harmfulness of e-cigarettes, most tweets denied that e-cigarettes were health hazards [34]. However, in our study, people were more concerned about the health problems and teenage vaping. Therefore, the public attitudes toward e-cigarettes became less positive over time, which might be due to the wide awareness of the potential health effects of e-cigarette use. There have been few studies on flavored e-cigarettes policy on social media. One study showed that although the flavored cigarette ban could be considered as successful in controlling adolescent tobacco use, there was a high probability that they would switch to other flavored tobacco products [3]. In addition, another study showed that after New York City banned flavored cigarettes, the sale of nonflavored tobacco products increased [35]. In our study, we showed that the proportion of negative e-cigarette tweets increased in both New York and other states, which might be due to the public awareness of the negative health effects of e-cigarettes or the potential effects of the New York State flavor policy.

### Limitations

In this study, we used Twitter data to analyze users’ attitudes toward e-cigarettes and the New York State policy on flavored e-cigarettes. Although Twitter is one of the most popular social media platforms in the United States, Twitter users might not represent the whole population as the demographic composition of Twitter users is different from that of the whole population. According to Pew Research Center data in 2018, approximately 24% of US adults used Twitter and 45% of the younger Americans (18-24 years old) were Twitter users [36]. Among adult Twitter users, only 15% regularly use Twitter, and young adults and minorities tend to be more highly represented on Twitter than in the general population. Meanwhile, very active and passive users are more prevalent than moderate users on Twitter. Thus, the results of this study were from a nonuniform subsample of tweets posted by a nonrepresentative portion of the US populations.

Other demographic information (including age, gender) were not included in our study owing to the limitation of Twitter data. In addition, the geographical location of users can be collected only if they are willing to share. Gore et al [37] mentioned that 95% of the Twitter users preferred not to share the location for a single tweet, and 1% of the users were willing to share the locations for the majority of the tweets they posted. However, in our data set, there were 68.10% (301,4419/4,426,290) of tweets containing the location of either tweets or users. Some tweets without geolocation information were not included in our study, which might introduce some biases. In addition, Padilla et al [38] showed that both temporal and spatial measures could bias the sentiment of an individual’s tweet. We did not examine the effect of temporal and spatial measures on the sentiment of the tweets, which might bias the sentiment results. In this study, we did not examine how the policy on flavored e-cigarettes affects the users’ behavior patterns such as switching to different flavored e-cigarettes, quitting vaping, or switching to cigarette smoking, which require further investigation. In addition, considering the co-occurrence of EVALI during our study period, there could be some biases with the potential impact of the New York flavor policy on public attitudes toward e-cigarettes.

In our study, we focused on analyzing the differences between public response to New York flavor policy in New York State and other states. Thus, we categorized the states that did not have a flavor policy as 1 group. However, this may cause limitations because these states may have their unique characteristics that might impact their attitudes toward New York flavor policy, such as their previous policies on tobacco products and government’s attitudes toward e-cigarettes. In addition, since San Francisco announced the flavor e-cigarette policy as a city, we only excluded the posts from that city. However, this policy might influence other cities in California, which could not be measured in this study.

Our results were insufficient for capturing the nuance of the conversation about flavored e-cigarette use. An increase in negative sentiments among policy-related tweets in New York State could reflect resistance toward additional regulation or concerns that banning flavored e-cigarettes might lead to increased cigarette usage among new and existing smokers. A machine learning classifier could be used in future studies to differentiate between individuals’ reasons for positive or negative perceptions toward the ban. Although the flavor policy on e-cigarettes in New York State was announced on September 17, 2019, it was never actually implemented in New York State during the study period. Therefore, the impact of the New York flavor policy might be underestimated. This might be one of the reasons that the changes in public attitudes toward e-cigarettes between New York State and other states were similar. The recent US Food and Drug Administration flavor enforcement policy implemented on February 6, 2020 and the New York State law on flavored vapor products, implemented on May 18, 2020, might have more obvious impact on public attitudes toward e-cigarettes, which will be explored in our future studies.

### Conclusions

Using social media data from Twitter, our study showed that after the policy on flavored e-cigarettes in New York State was announced, the discussions about e-cigarettes and the flavor policy increased significantly. Twitter users in the states that did not have a flavored e-cigarette policy have similar concerns about the flavor policy as those in New York State. Sentiment analysis revealed that after the New York flavor policy was announced, the public tended to have a more negative attitude toward e-cigarettes in New York State and other states. Together, our study provides an initial investigation about the potential impact of the New York State policy of flavored e-cigarettes on the public attitudes toward e-cigarettes, which might subsequently affect user behavior.

## Abbreviations

e-Cigarette: electronic cigarette
EVALI: e-cigarette or vaping use–associated lung injury
PATH: Population Assessment of Tobacco and Health

## References

[1] King BA, Tynan MA, Dube SR, Arrazola R (2014). Flavored-little-cigar and flavored-cigarette use among U.S. middle and high school students. J Adolesc Health.

[2] Villanti AC, Richardson A, Vallone DM, Rath JM (2013). Flavored tobacco product use among U.S. young adults. Am J Prev Med.

[3] Courtemanche CJ, Palmer MK, Pesko MF (2017). Influence of the Flavored Cigarette Ban on Adolescent Tobacco Use. Am J Prev Med.

[4] Gentzke AS, Creamer M, Cullen KA, Ambrose BK, Willis G, Jamal A, King BA (2019). Vital Signs: Tobacco Product Use Among Middle and High School Students - United States, 2011-2018. MMWR Morb Mortal Wkly Rep.

[5] Banning the sale of flavored tobacco products. City and County of San Francisco Ordinance.

[6] Grana R, Benowitz N, Glantz SA (2014). E-Cigarettes. Circulation.

[7] Schneller LM, Bansal-Travers M, Goniewicz ML, McIntosh S, Ossip D, O'Connor Richard J (2019). Use of Flavored E-Cigarettes and the Type of E-Cigarette Devices Used among Adults and Youth in the US-Results from Wave 3 of the Population Assessment of Tobacco and Health Study (2015-2016). Int J Environ Res Public Health.

[8] Gerloff J, Sundar IK, Freter R, Sekera ER, Friedman AE, Robinson R, Pagano T, Rahman I (2017). Inflammatory Response and Barrier Dysfunction by Different e-Cigarette Flavoring Chemicals Identified by Gas Chromatography-Mass Spectrometry in e-Liquids and e-Vapors on Human Lung Epithelial Cells and Fibroblasts. Appl In Vitro Toxicol.

[9] Gotts JE, Jordt S, McConnell R, Tarran R (2019). What are the respiratory effects of e-cigarettes?. BMJ.

[10] Lee WH, Ong S, Zhou Y, Tian L, Bae HR, Baker N, Whitlatch A, Mohammadi L, Guo H, Nadeau KC, Springer ML, Schick SF, Bhatnagar A, Wu JC (2019). Modeling Cardiovascular Risks of E-Cigarettes With Human-Induced Pluripotent Stem Cell-Derived Endothelial Cells. J Am Coll Cardiol.

[11] Krishnasamy Vikram P, Hallowell Benjamin D, Ko J, Board Amy, Hartnett Kathleen P, Salvatore Phillip P, Danielson Melissa, Kite-Powell Aaron, Twentyman Evelyn, Kim Lindsay, Cyrus Alissa, Wallace Megan, Melstrom Paul, Haag Brittani, King Brian A, Briss Peter, Jones Christopher M, Pollack Lori A, Ellington Sascha, Lung Injury Response Epidemiology/Surveillance Task Force (2020). Update: Characteristics of a Nationwide Outbreak of E-cigarette, or Vaping, Product Use-Associated Lung Injury - United States, August 2019-January 2020. MMWR Morb Mortal Wkly Rep.

[12] Siegel D (2019). Update: Interim Guidance for Health Care Providers Evaluating and Caring for Patients with Suspected E-cigarette, or Vaping, Product Use Associated Lung Injury. Morbidity and Mortality Weekly Report.

[13] Restricting the sale, manufacture, and distribution of tobacco products, including electronic cigarettes. City and County of San Francisco.

[14] Michigan’s ban of flavored nicotine vaping products to protect children’s health effective immediately with rules filing. MDHHS.

[15] Governor Cuomo announces New York State implements first-in-the-nation ban on flavored e-cigarettes. CBS News.

[16] Emergency notice of changes in regulations that may affect your business. Rhode Island Department of Health.

[17] Tobacco control and prevention program. County of Los Angeles Public Health.

[18] OHA, OLCC file rules banning flavored vaping sales, including online. Oregon.gov.

[19] Governor Bullock directs ban on flavored e-cigarettes to address public health emergency. Great Falls Tribune.

[20] Washington State bans vapor products containing vitamin E acetate. Washington State Department of Health.

[21] Blumenfeld K (2015). Electronic smoking devices. GASP.

[22] Minimum standards for retail sale of tobacco and electronic nicotine delivery systems. Department of Public Health.

[23] Kavuluru R, Sabbir A (2016). Toward automated e-cigarette surveillance: Spotting e-cigarette proponents on Twitter. J Biomed Inform.

[24] Zhou J, Zhang Q, Zeng DD, Tsui KL (2018). Influence of Flavors on the Propagation of E-Cigarette-Related Information: Social Media Study. JMIR Public Health Surveill.

[25] Chu K, Allem J, Unger JB, Cruz TB, Akbarpour M, Kirkpatrick MG (2019). Strategies to find audience segments on Twitter for e-cigarette education campaigns. Addict Behav.

[26] Wojcik S, Hughes A Sizing up Twitter users. Pew Research Center.

[27] Schober MF, Pasek J, Guggenheim L, Lampe C, Conrad FG (2016). Social Media Analyses for Social Measurement. Public Opin Q.

[28] Farrimond H (2016). E-cigarette regulation and policy: UK vapers' perspectives. Addiction.

[29] Lienemann BA, Unger JB, Cruz TB, Chu K (2017). Methods for Coding Tobacco-Related Twitter Data: A Systematic Review. J Med Internet Res.

[30] Chen L, Lu X, Yuan J, Luo J, Luo J, Xie Z, Li D (2020). A Social Media Study on the Associations of Flavored Electronic Cigarettes With Health Symptoms: Observational Study. J Med Internet Res.

[31] Lu X, Chen Long, Yuan Jianbo, Luo Joyce, Luo Jiebo, Xie Zidian, Li Dongmei (2020). User Perceptions of Different Electronic Cigarette Flavors on Social Media: Observational Study. J Med Internet Res.

[32] Hutto C, Gilbert E (2015). VADER: A parsimonious rule-based model for sentiment analysis of social media text. Proceedings of the International AAAI Conference on Web and Social Media.

[33] Blei D, Ng AY, Jordan MI (2003). Latent dirichlet allocation. Journal of Machine Learning Research.

[34] Martinez LS, Hughes S, Walsh-Buhi ER, Tsou M (2018). "Okay, We Get It. You Vape": An Analysis of Geocoded Content, Context, and Sentiment regarding E-Cigarettes on Twitter. J Health Commun.

[35] Farley SM, Johns M (2017). New York City flavoured tobacco product sales ban evaluation. Tob Control.

[36] Smith A, Anderson M Social media use in 2018. Pew Research Center.

[37] Gore RJ, Diallo S, Padilla J (2015). You Are What You Tweet: Connecting the Geographic Variation in America's Obesity Rate to Twitter Content. PLoS One.

[38] Padilla JJ, Kavak H, Lynch CJ, Gore RJ, Diallo SY (2018). Temporal and spatiotemporal investigation of tourist attraction visit sentiment on Twitter. PLoS One.

